# Insight into the Lifestyle of Amoeba *Willaertia magna* during Bioreactor Growth Using Transcriptomics and Proteomics

**DOI:** 10.3390/microorganisms8050771

**Published:** 2020-05-21

**Authors:** Issam Hasni, Philippe Decloquement, Sandrine Demanèche, Rayane Mouh Mameri, Olivier Abbe, Philippe Colson, Bernard La Scola

**Affiliations:** 1 Aix-Marseille University, Institut de Recherche pour le Développement IRD 198, Assistance Publique—Hôpitaux de Marseille (AP-HM), Microbes, Evolution, Phylogeny and Infection (MEΦI), UM63, 13005 Marseille, France; issemhasni@gmail.com (I.H.); Philippe.DECLOQUEMENT@univ-amu.fr (P.D.); philippe.colson@univ-amu.fr (P.C.); 2 R&D Department, Amoéba, 69680 Chassieu, France; sandrine.demaneche@amoeba-biocide.com (S.D.); mameri77@yahoo.fr (R.M.M.); Olivier.Abbe@amoeba-biocide.com (O.A.); 3 Institut Hospitalo-Universitaire (IHU)—Méditerranée Infection, 13005 Marseille, France

**Keywords:** *Willaertia magna*, amoeba, environmental, ecological, bioreactor, transcriptomics, proteomics, biocide

## Abstract

*Willaertia magna* C2c maky is a thermophilic free-living amoeba strain that showed ability to eliminate *Legionella pneumophila*, a pathogenic bacterium living in the aquatic environment. The amoeba industry has proposed the use of *Willaertia magna* as a natural biocide to control *L. pneumophila* proliferation in cooling towers. Here, transcriptomic and proteomic studies were carried out in order to expand knowledge on *W. magna* produced in a bioreactor. Illumina RNA-seq generated 217 million raw reads. A total of 8790 transcripts were identified, of which 6179 and 5341 were assigned a function through comparisons with National Center of Biotechnology Information (NCBI) reference sequence and the Clusters of Orthologous Groups of proteins (COG) databases, respectively. To corroborate these transcriptomic data, we analyzed the *W. magna* proteome using LC–MS/MS. A total of 3561 proteins were identified. The results of transcriptome and proteome analyses were highly congruent. Metabolism study showed that *W. magna* preferentially consumed carbohydrates and fatty acids to grow. Finally, an in-depth analysis has shown that *W. magna* produces several enzymes that are probably involved in the metabolism of secondary metabolites. Overall, our multi-omic study of *W. magna* opens the way to a better understanding of the genetics and biology of this amoeba.

## 1. Introduction

Most free-living amoebas (FLAs) are classified in two suprakingdom-level groups: *Amoebozoa* including *Acanthamoeba* spp. or *Vermamoeba* spp. and *Excavata* comprising *Naegleria* spp., *Vahlkampfia* spp. or *Willaertia* spp. [1,2]. *Willaertia magna* is a FLA belonging to family *Vahlkampfiidae* [3]. This protist presents a three-stage life, switching between a trophozoite form, a flagellate form and a cystic form [4,5]. The *W. magna* trophozoite (50–100 µm in size) form corresponds to the main active stage during which this thermophilic amoeba feeds, moves and divides; it can grow at various temperatures ranging from 22 to 44 °C in xenic or axenic culture. Under unfavorable conditions, the amoeba adopts a cystic form (18–21 µm in size), which allows its survival until the conditions become favorable for cell growth. The transformation to the flagellate stage occurs temporally, being triggered by various stressors [3].

FLAs graze on microorganisms to grow, and this regulates the microbial population in the environment [6,7]. However, some bacteria, such as *Legionella pneumophila*, have evolved to bypass the mechanism of elimination in order to survive and multiply within amoebae [8,9,10]. *L. pneumophila* is a human pathogen causing legionellosis, a disease transmitted by the inhalation of contaminated aerosol [11]. This Gram-negative bacterium is ubiquitous in natural and man-made fresh water environments such as cooling towers [12,13]. It has the ability to infect and invade a wide range of amoebae, including *Vermamoeba*, *Acanthamoeba* and *Naegleria* species [14]. Furthermore, the amoeba cysts serve as protection for *L. pneumophila* in adverse conditions making legionella management in cooling tower water using chemical biocides difficult [15,16]. Amoebae can adopt different behaviors with respect to *L. pneumophila*. If *Vermamoeba* and *Acanthamoeba* appear as highly susceptible to a wide range of *Legionella* spp., previous studies reported the elimination of pathogenic *L. pneumophila* strain Paris 1 (ATCC 33152) by *W. magna* C2c maky [17,18,19,20]. The Amoéba^®^ company developed a natural biocide based on the property of *W. magna* C2c maky to control *L. pneumophila* proliferation and as an alternative to chemical biocides in cooling tower water [21]. To treat cooling tower water with this natural biocide, it is necessary to produce a large number of amoebae in a short time. However, traditional methods based on axenic amoebal cultivation in adhesion [4,22] do not provide high enough cell densities in a short time [23]. Therefore, high-throughput production of cells in bioreactor culture is a strategy developed to improve the productivity [24]. This process is usually used for the mass production of bacteria, fungi or microalgae of pharmaceutical interest [25,26,27].

In a previous study, the analysis of the *W. magna* genome indicated a size of 36 megabase pairs (Mbp) and a set of 18,561 genes [28]. The gene content survey included a detailed analysis of genes potentially involved in pathogenicity and of sequence transfers with pathogenic microorganisms. This study failed to detect virulence factors and showed the non-pathogenicity in vitro of *W. magna* [28]. Furthermore, we observed putative exchanges for 136 genes belonging to amoeba-resistant microorganisms [28].

To expand our knowledge on *W. magna* C2c maky, we explored the transcriptome as well as the proteome of this amoeba. RNA-seq is an effective method to understand and identify the level of expression of genes involved in the different metabolic pathways and biochemical processes [29]. It is accurately complemented by proteome analysis for these goals [30,31]. Furthermore, we compared our results with *Naegleria gruberi* data [32,33], a non-pathogenic amoeba that is phylogenetically close to *W. magna*, and with data on *Acanthamoeba castellanii*, which is the most studied FLA due to its interest regarding human health and its potential role in the ecosystem [34,35,36].

## 2. Materials and Methods

### 2.1. W. magna C2c maky Culture in Bioreactor

*W. magna* C2c maky cells (ATCC^®^ PTA-7824) were precultured in SCGYEM medium [37] supplemented with 10% calf serum contained in 175 cm^2^ culture flask at 37 °C. Exponentially growing cells were harvested and used to inoculate a 10-L bioreactor (GPC, La Rochelle, France; [38]) in SCGYEM medium without fetal calf serum (pH = 7) at a temperature varying between 30 and 45 °C under helix constant agitation (200 rpm) and oxygenation. The amoeba cells were harvested from a bioreactor at a volume of 50 mL and were centrifugated at 2000 x g for 10 min followed by three steps of washing using Page’s modified Neff’s Amoeba Saline medium (2 mMNaCl, 16 μM MgSO4, 27.2 μM CaCl2, 1 mM Na2HPO4, 1 mM KH2PO4). Amoeba quantification was performed using a KOVA^®^ slide cell counting chamber (KOVA International, CA, USA).

### 2.2. Illumina Sequencing of the Transcriptome

The total RNA of each sample was extracted using a RNeasy Mini Kit (Qiagen, Hilden, France) according to the manufacturer’s instructions. RNA was quantified and qualified using a QuantiFluor RNA sample system (Promega, Charbonnières, France) and a nano RNA chip on a BioAnalyser 2100 (Agilent Technologies Inc., Santa Clara, CA, United States), respectively. Illumina sequencing was performed using ProfileXpert/Viroscan3D, Lyon. Briefly, libraries were prepared from total RNA using poly(A) enrichment of the mRNA to remove ribosomal RNA. NextFlex rapid directional RNAseq sample prep (Bioo Scientific Corporation) was used to achieve the libraries. Quantification and validation of the libraries were performed using dsDNA HS Assay on a Quantus Fluorometer (Promega, Charbonnières, France) and on a BioAnalyser 2100 from Agilent using a HS DNA chip (Agilent Technologies Inc., Santa Clara, CA, United States), respectively. The library was sequenced in 75 base pair (bp) length paired end reads in NextSeq 500 Mid Output flow cell lines from Illumina (Illumina Inc, San Diego, CA, USA) generating 216.9 million raw read pairs.

### 2.3. Transcriptomic Analysis of W. Magna C2c Maky

The quality assessment of raw reads was checked using FastQC software (https://www.bioinformatics.babraham.ac.uk/projects/fastqc/) for reads with low quality (Q < 28), and the adaptors were removed using Trimmomatic [39]. The identification of transcripts was performed as in the study conducted by Aherfi et al. [40]. Briefly, reads were mapped on the *W. magna* C2c maky assembled genome using Hisat2 with default parameters [41]. The expression of genes was quantified using Htseq [42], a software that processes data from high-throughput sequencing assays. This software identifies and counts the number of reads mapped to each gene. Predicted open reading frames (ORF) with a coverage of six reads or more were considered as transcribed [40]. This threshold was determined in order to obtain transcripts with high coverages (61 reads/ORFs).

### 2.4. Functional Annotation

To assign the biological functions of *W. magna* transcripts, we proceeded to homology searches for the transcripts in public protein databases. Predicted protein sequences were used as queries against the nr database using an e-value threshold of less than 1e-03. Protein sequences were aligned to the Cluster Orthologous Groups of proteins (COG) database using EggNOG [43,44] with diamond as mapping mode. To improve understanding of the biological functions and metabolic pathways of the genes, the sequences were mapped on Kyoto Encyclopedia of Genes and Genomes Pathway (KEGG) [45] using BLASTp with an e-value cutoff of 1e-3. Conserved protein domains were identified by mapping of proteins against the conserved domain database [46] and InterProScan database [47]. The nonribosomal peptide synthase and polyketide synthase domains were predicted using 2metDB [48] and NaPDoS [49] software. To predict the localization of proteins in the cell, we used TargetP 2.0 and MitoP2 [50,51]. To perform an analysis of genes related to anaerobic metabolism, we performed a BLASTp search against a non-redundant protein sequences database (nr), with an e-value cutoff of 1e-03. Alignment of protein sequences was carried out using MUSCLE [52]. Phylogenetic construction was obtained using the maximum likelihood method with the Jones–Taylor–Thornton (JTT) model on MEGA 7.0.25 software [53]. Phylogenetic trees were analyzed using iTOL v3 online [54].

### 2.5. Proteomics

#### 2.5.1. Cell Lysis

Two samples of *W. magna* C2c maky obtained from two independent bioreactors were used in the proteomic study. *W. magna* pellets, containing 5 × 10^8^ cells, were lysed in 2 mL of buffer 1X phosphate buffered saline (pH 8.0) and 1% sodium dodecyl sulfate (SDS) by gram of pellet for 2 h at 4 °C under agitation. Lysates were then centrifuged at 3000× *g* for 15 min at 4 °C to remove cellular debris, and the supernatants were stored at 4 °C. Total protein concentration in lysate fractions was determined using the Pierce micro bicinchoninic acid (BCA) protein assay kit (Thermo Fisher Scientific, Illkirch, France) by using bovine serum albumin (BSA) as a standard.

#### 2.5.2. SDS-Page and Western-Blot

Protein lysate samples were denatured and reduced in SDS/TCEP-loading buffer (Genentech, Inc., So. San Francisco, CA, US) and separated on a 4%‒15% acrylamide gel (4%–15% Criterion^®^ TGX Stain-Free™ Gel, Bio-Rad, Hercules, CA, US). Migration was performed in Tris- Glycine-SDS buffer (Euromedex, Souffelweyersheim, France). The acrylamide gel was then activated under UV light with a ChemiDoc™ MP system (Bio-Rad, Hercules, CA, US) for Stain-Free™ detection. The SDS-PAGE gel was subsequently Coomassie-stained for 1 h with InstantBlue Ultrafast Protein Stain (Sigma-Aldrich Chimie S.a.r.l. Lyon, France) and proteins were visualized with a ChemiDoc™ MP system (Bio-Rad, Hercules, CA, US).

#### 2.5.3. Liquid Digestion

Following the protein precipitation step (using trichlororoacetic acid 20% in volume, overnight at 4 °C), samples were washed in acetone twice and solubilized in 8 M urea. Then, samples were reduced (Tris(2-CarboxyEthyl)Phosphine, 5 mM, 57 °C, 1h), alkylated (iodoacetamide, 10 mM, RT, 45 min), and digested for 5 h at 37 °C with LysC and overnight at 37 °C with trypsin (1/100 ratio). Peptides digest was next fractionated on a high pH reversed phase fractionation spin column (Thermo Scientific) according to the manufacturer’s instructions. The 8 fractions obtained were dried in a speed vacuum before nanoLC-MS/MS analysis and then suspended in 40 µL 0.1% HCOOH.

#### 2.5.4. NanoLC-MS/MS Analysis

The fractions were analyzed using an Ultimate 3000 nano-RSLC (Thermo Scientific, San Jose California) coupled online with a Q Exactive HF mass spectrometer via a nano-electrospray ionization source (Thermo Scientific, San Jose California).

2 µL of peptide mixtures were loaded on a C18 PepMap100 trap-column (75 µm i.d. × 2 cm, 5 µm, 100Å, Thermo Fisher Scientific) for 3 min at 5 µL/min with 2% ACN, 0.05% TFA in H_2_O and then separated on a C18 Acclaim PepMap100 nano-column, 50 cm × 75 µm i.d, 2 µm, 100 Å (Thermo Scientific) with a 60 min linear gradient from 3.2% to 40% buffer B (A: 0.1% FA in H_2_O, B: 0.1% FA in ACN) and then from 40% to 90% of B in 2 min, held for 10 min and returned to the initial conditions in 1 min for 15 min. The total duration was set to 90 min at a flow rate of 300 nL/min. The oven temperature was kept constant at 40 °C.

The sample was analysed with the TOP20 HCD method: MS data were acquired in a data dependent strategy selecting the fragmentation events based on the 20 most abundant precursor ions in the survey scan (350-1600 Th). The resolution of the survey scan was 60,000 at m/z 200 Th. The Ion Target Value for the survey scans in the Orbitrap and the MS^2^ mode were set to 3E6 and 1E5 respectively and the maximum injection time was set to 60 ms for both scan modes. Parameters for acquiring HCD MS/MS spectra were as follows: collision energy = 27 and an isolation width of 2 m/z. The precursors with unknown charge state or a charge state of 1 were excluded. Peptides selected for MS/MS acquisition were then placed on an exclusion list for 20 s using the dynamic exclusion mode to limit duplicate spectra.

### 2.6. Data Analysis

The data were converted to mgf format using RawConverter [55]. For each sample, the 8 fractions were merged into a single dataset and the resulting peak lists were searched against the protein sequences of *W. magna* C2c maky using Peaks Studio software (Bioinformatics Solutions Inc, Waterloo, Canada) [56]. The search was performed using the following settings, based on the *W. magna* genome database (18,519 sequences): peptide mass error tolerance, 25 ppm; fragment mass error tolerance, 0.1 Da; monoisotopic mass values, one missed cleavage, no non-specific cleavage, fixed modifications of carbamidomethylation; variable modifications of oxidation (M), deamidation (NQ), carbamylation and oxidation or hydroxylation; 4 maximum variable post-translational modifications (PTMs) per peptide. The peptides identified were filtered based on an FDR (false discovery rate) cut-off of 0.5%.

## 3. Results

### 3.1. Transcriptomic Analysis

RNA sequencing generated 217 million raw reads in total with 75 bp for both paired ends. After removal of adaptor sequences and improving the stringency quality, we obtained 14 gigabases of cleaned data, and 49,015,843 nucleotides (nt) were generated. To analyze gene expression, the reads were mapped against the *W. magna* C2c genome, resulting in 86.1% of reads mapped. The expression of the *W. magna* genes was examined and 8,790 *W. magna* C2c transcripts (47.4% of *W. magna* ORFs) were detected. Among the 8,790 transcripts, BLASTp analysis against the nr database found homologous sequences for 6,179 (70.3%) of the transcribed genes and identified 2,611 ORFans among the transcript genes (29.7%) (Table S1). Of the ten genes with the highest coverage (mean coverage of 4,316 reads/gene), two transcripts were assigned to a function (bactericidal permeability-increasing protein/lipopolysaccharide-binding protein and histidine triad motif (HIT) family protein), five were annotated as hypothetical proteins and two as ORFans (Table S1). To further analyze the most highly expressed genes, we studied the conserved domains of hypothetical proteins and ORFans. Among the five hypothetical proteins, we found a conserved domain for three predict genes, including a glutathione S-transferase domain, a WD40 domain and a flavoprotein domain (Table S1). For one of two ORFs with no match in the nr database, we identified one F-box domain (Table S1).

### 3.2. Mean Proteomic Information

The RNA-seq analysis was supplemented with a proteomic analysis carried out on two biological replicates of *W. magna* grown in the bioreactor.

**Figure 1:**
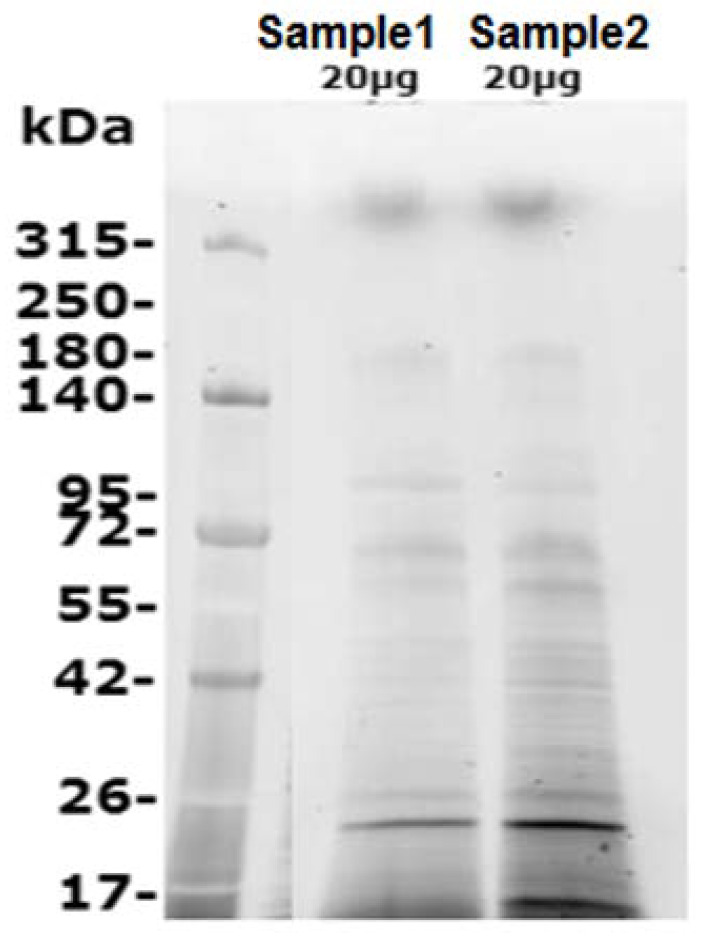
Detection of amoebal proteins using SDS-PAGE analysis. After separation on SDS-PAGE gel, proteins were visualized using stain-free^TM^ detection sample 1 and sample 2: total proteins.

The lysate concentrations of replicates 1 and 2 were estimated using the micro BCA method at 11.6 and 15.6 mg/mL, respectively. A total of 20 μg of each sample were separated on SDS-PAGE gel and proteins were visualized using Stain-Free™ detection (Figure 1).

**Figure 2:**
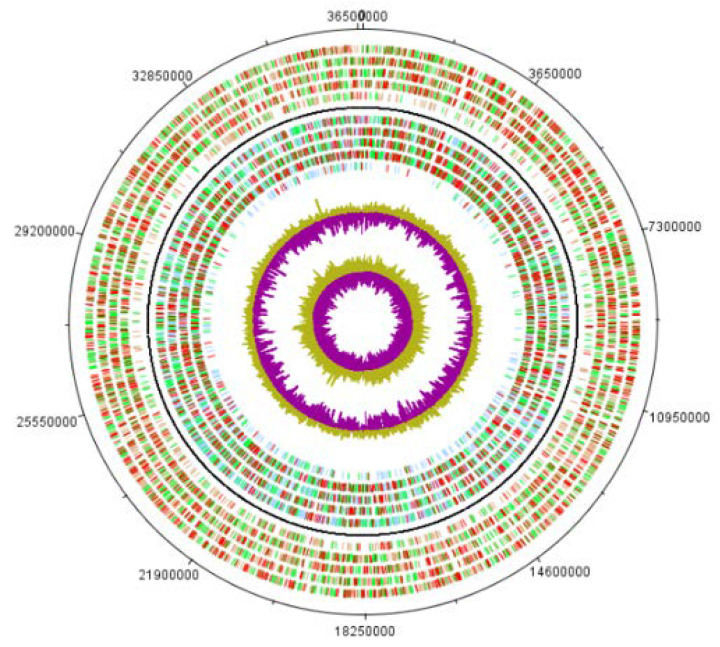
Circular map of the *W. magna* genome. Outside circle: genes on the forward strand colored by light brown are predicted genes; green bars are transcripts and red are proteins. Second to outside circle: genes on the reverse strand colored by light blue are predicted genes; green bars are transcripts and red bars are proteins. In the center: GC content and GC skew.

Expressed proteins were identified using the Peaks Studio program (Table S2) [57]. A total of 106,383 spectra along with 64,260 peptides were identified. In total, we detected 3561 proteins, which represented 19.2% of the predicted genes and 40% of the transcriptome (Figure 2 and Table S1).

As we processed the proteomic analysis twice, we detected 2970 proteins from the first analysis and 3186 proteins from the second one (Table S2). The annotation using BLASTp against the National Center of Biotechnology Information (NCBI) protein sequence database assigned a putative function to 3376 proteins, among which 3013 had as best hits proteins belonging to *N. gruberi* and one best matched with a giant virus (*Moumouvirus* Monve) protein. The taxonomical distribution revealed a very high proportion of genes shared with eukaryotes (97.9%), followed by genes shared with bacteria (1.9%), archaea (0.15%) and viruses (0.03%) (Figure S1).

### 3.3. COG Enrichment Analysis

**Figure 3:**
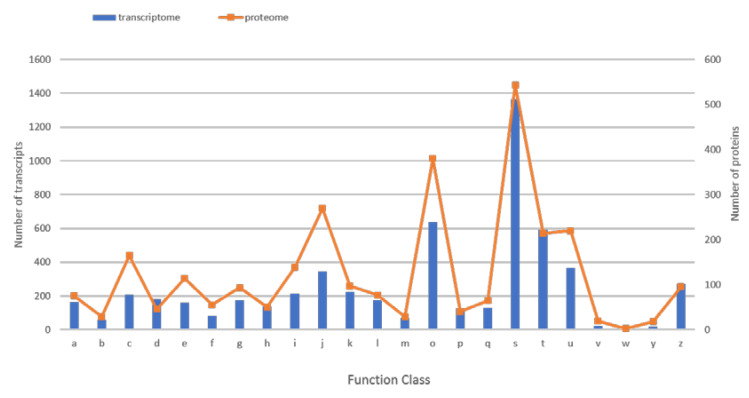
Representation of *W. magna* transcripts and proteins related to different functional categories in COG database. A: RNA processing and modification, B: Chromatin structure and dynamics, C: Energy production and conversion, D: Cell cycle control, cell division, chromosome partitioning, E: Amino acid transport and metabolism, F: Nucleotide transport and metabolism, G: Carbohydrate transport and metabolism, H: Coenzyme transport and metabolism, I: Lipid transport and metabolism, J: Translation, ribosomal structure and biogenesis, K: Transcription, L: Replication, recombination and repair, M: Cell wall/membrane/envelope biogenesis, O: Posttranslational modification, protein turnover, chaperones, P: Inorganic ion transport and metabolism, Q: Secondary metabolites biosynthesis, transport and catabolism, S: Function unknown, T: Signal transduction mechanisms, U: Intracellular trafficking, secretion and vesicular transport, V: Defense mechanisms, W: Extracellular structures, Y: Nuclear structure, Z: Cytoskeleton.

To further analyze the function of the transcripts and proteins, we conducted a comparison of the latter with the COG database (Figure 3).

A total of 5341 (60.7%) and 2723 (76.5%) transcripts and proteins, respectively, were assigned to COG categories (Table S1). Overall, these transcripts and proteins were distributed in 23 COG categories. Regarding transcripts, the category “function unknown” (1366: 25%) was the most represented, followed by “post-translational modification, protein turnover, chaperones” (636: 12%), “signal transduction mechanisms” (593: 11%), “intracellular trafficking, secretion and vesicular transport” (366: 7%) and “translation, ribosomal structure and biogenesis” (346: 6%). Regarding proteins, the COG category in which the greatest number of proteins were assigned is the category “function unknown” (543: 20%) followed by “post-translational modification, protein turnover, chaperones” (380: 14%), “translation, ribosomal structure and biogenesis” (270: 10%), “intracellular trafficking, secretion and vesicular transport” (220: 8%) and “signal transduction mechanisms” (214: 8%). Categories “nuclear structure” (18: 0.3% transcripts and 18: 0.7% proteins) and “extracellular structure” (10: 0.2% and 3: 0.1% proteins) were weakly represented among *W. magna* transcripts and proteins. Thus, the COG analysis revealed a similar top five categories for transcripts and proteins.

### 3.4. KEGG Enrichment Analysis

**Figure 4:**
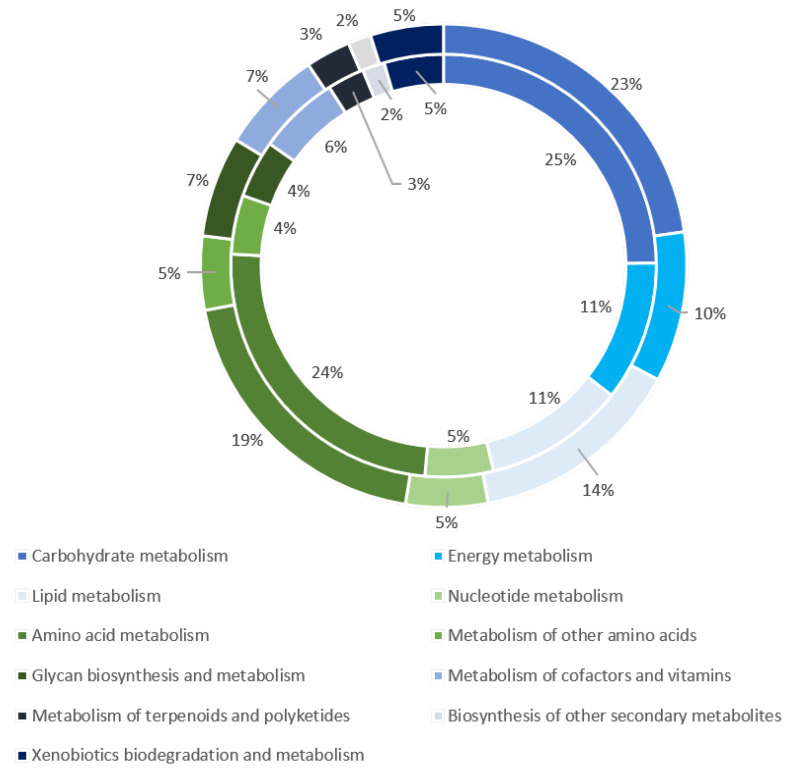
Representation of the *W. magna* transcripts and proteins related to metabolism. The transcripts and proteins are represented by the outer ring and inner ring, respectively.

An enrichment analysis with KEGG pathways was performed in order to get a better insight into the biological function and pathways of the expressed genes and proteins. Particularly, the KEGG study made it possible to identify the proteins involved in biochemical metabolism, the signal transduction pathway and genetic information processing. There were 3341 transcripts and 1999 proteins mapped into 368 and 362 KEGG pathways, respectively. Among them, 1173 transcripts and 846 proteins were assigned to metabolic pathways classified into 11 groups (Figure 4).

The map with the greatest number of transcripts and proteins was the carbohydrate pathway (267 transcripts and 220 proteins), followed by amino acid metabolism (229 transcripts and 217 proteins), lipid metabolism (167 transcripts and 95 proteins) and energy metabolism (118 transcripts and 95 proteins). This study identified various numbers of transcripts and proteins involved in glycolysis, pyruvate metabolism, lipid degradation and amino acid degradation (Table S3). These metabolisms seemed to be the main pathways used by the amoeba to provide the pyruvate for the Krebs cycle and energy cells. Moreover, analysis of KEGG pathways revealed that 18 transcripts were classified into 10 sub-categories belonging to a secondary metabolite category, including genes involved in the streptomycin and penicillin biosynthesis. The latter were also found at the proteomic level. In addition, we found that 755 genes and 527 proteins matched to genetic information processing, involving transcription, translation, folding, sorting, degradation, replication and repair. Finally, KEGG analysis identified 1408 genes and 773 proteins classified into membrane transport, signal transduction and environmental adaptation.

### 3.5. W. magna Shape and Movement

The investigation on *W. magna* motility revealed the presence of 349 transcripts and 151 proteins related to the cytoskeleton (Table S4). As for in silico analyses for *N. gruberi* and *A. castellanii* previously published, the cytoskeleton of *W. magna* seems to be mainly composed of microtubules and actin filaments [32,58]. Among the genes related to actin, we identified actin proteins that are the globular components of the microfilament (Table S4). In addition, we found actin-related proteins and myosin, which regulates actin microfilaments through its interaction with actin proteins [58] (Table S4). We detected components involved in microfilament organization including a Rho-family protein (Ras homolog family member) (Table S4). RhoA activates diaphanous-related formins, thereby contributing to the stimulation of actin polymerization [59]. The cell motility and cellular transport of *W. magna* are regulated by microtubules. Coherently, we found the presence of several transcripts (132) and proteins (31) related to microtubules, such as tubulins or motor proteins (dynein and kinesin). The association of protein motors and microtubules plays a critical role in intracellular organelle transport and is involved in the maintenance of cell shape [58]. We also detected microtubule proteins that make up the internal structure of the flagella (Table S4). In addition to genes related to cytoskeleton, some specific genes related to flagellar formation were identified (cilia- and flagella-associated protein 43, cilia- and flagella-associated protein 74 and long flagella protein lf4). This might suggest that *W. magna* is temporarily present in its flagellate form during suspension culture, but such form could not be observed using microscopy (unpublished data). Among the genes related to movement, we found that the genes encoding long flagella protein lf4, zinc finger, regulator of chromosome condensation, tubulin and dynein were the most expressed (Table S4).

### 3.6. Metabolism

The analysis of *W. magna* metabolism enabled a set of enzymes involved in glycolysis to be identified (Table S5). The presence of glucokinase, ribokinase and fructokinase revealed that the *W. magna* is able to use glucose and also several other monosaccharides for this carbohydrate requirement. *W. magna* shares some similarities with *N. gruberi* regarding the glycolysis pathway [33]. Indeed, *W. magna* does not have a hexokinase, an enzyme involved in the first step of glycolysis for the phosphorylation of all hexoses. Nevertheless, we reported the expression of a hexokinase homolog (glucokinase), which is a glucose-specific enzyme. Furthermore, the phosphorylation of fructose 6-phosphate in the second step of glycolysis is not carried out by classical ATP-dependent phosphofructokinase (PFK) but is catalyzed by a pyrophosphate-fructose 6-phosphate 1-phosphotransferase (ppi-PKF). The hexose-monophosphate pathway is another metabolic pathway used by *W. magna*, which degrades the glucose in pyruvate. Moreover, we detected all the enzymes involved in the consumption of glycerol that may serve as an energy substrate (Table S5). Finally, we identified components involved in the Krebs cycle and mitochondrial respiratory chain. *W. magna* is an aerobic organism that uses oxygen as terminal acceptor. However, we reported the presence of a nitrate reductase, which is an enzyme involved in anaerobic metabolism (Table S5) [32,33]. Furthermore, our analysis revealed the presence of FeFe-hydrogenase and one associated maturase (FeFe-H2ase maturase proteins HydG). These enzymes are involved in energy metabolism for microaerophilic or anaerobic microorganisms producing molecular hydrogen [35,60]. Of these three proteins, two seemed to have N-terminal mitochondrial transit peptides (Table S5). These three proteins produced by *W. magna* shared best hits with *Naegleria gruberi* proteins. The phylogenetic reconstructions based on these genes related to anaerobic respiration showed a clustering with their homologs in *Naegleria* organisms (Figures S2–S4). All these genes related to anaerobic respiration were weakly expressed (Table S5).

### 3.7. Defense Mechanisms

**Figure 5:**
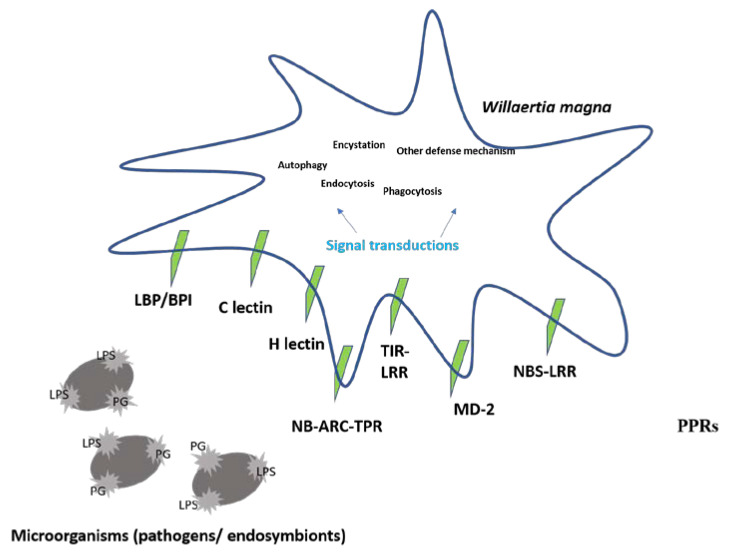
Mechanism of pattern recognition and pattern-recognition receptors (PRRs) identified within *Willaertia magna*: Lipopolysaccharide (LPS), Proteoglycan (PG), Lipopolysaccharide binding protein/Bactericidal Permeability-Increasing protein (LPS/BPI), C-lectin, H-lectin, Nucleotide-Binding Adaptor R-gene and Tetratricopeptide Repeat (NB-ARC-TPR), Toll/Interleukin-1 Receptor and Leucine-Rich Repeat (TIR-LRR domain), Nucleotide Binding Site and Leucine Rich Repeat (NBS-LRR domain) and MD-2.

To characterize the defense mechanisms of *W. magna*, we searched for the presence of predicted pattern-recognition receptors (PRRs) (Figure 5 and Table S6).

*W. magna* contains three members of the bactericidal permeability-increasing protein/lipopolysaccharide-binding protein family and a MD-2-related protein. All these four receptors play an important role in triggering defensive responses through their interactions with lipopolysaccharide of bacteria [61]. This gene shared a best hit in the nr database with a *N. gruberi* sequence. Furthermore, we reported the presence of C- and H-lectin domains (three) that belong to a large family of receptors that bind carbohydrates and induce endocytic, phagocytic and antimicrobial responses [35] (Table S6). Four mannose-binding proteins were revealed within the *W. magna* transcriptome. Among them, two best matched with *N. gruberi*, one with *Tetrahymena thermophila* and one with *Rathayibacter tanaceti*. These glycoproteins are involved in the endocytosis of *L. pneumophila* by *A. castellanii* [34]. In *A. castellanii* and *Dictyostelium discoideum*, leucine-rich repeat (LRR) containing nucleotide-binding adaptor R-gene (NBARC) tetratricopeptide repeat (TPR) or Toll/interleukin-1 receptor (TIR) domains are assumed to be involved in the immune responses [35,62]. *W. magna* was predicted here to possess 18 LRR-containing proteins according to its transcriptome; 17 have a transmembrane domain, two are homologs of a NBARC-TPR, one is homologous to a nucleotide binding site and leucine rich repeat (NBS-LRR) domain and one is homologous to a TIR domain, such as those found in Toll-like receptors. Although *W. magna* is produced in high-throughput axenic culture, we identified the genes involved in the bacterial degradation. Indeed, we identified a homologous of Nox2, an important protein of the NADPH-oxidase membrane-bound enzyme complex that is implicated in the oxidative burst of phagocytic cells and plays a role in bacterial killing by free radicals [63]. This gene was highly transcribed, but we had not identified this protein within proteome data. The *W. magna* also displayed a large range of enzymes involved in the hydrolysis of carbohydrates, such as chitinases, lysozymes, β-hexosaminidases and amylase. In addition, we reported the presence of different isoforms of cathepsins, a group of lysosomal hydrolases involved in protein degradation [64,65]. Finally, we found transcripts and proteins related to the phago-lysosomal system (phospholipases and lipases), which is consistent with the capability of *W. magna* to digest bacteria (Table S6).

*W. magna* possesses the essential weapons to deal with microorganism attacks. However, all the genes identified in bacterial defense and destruction are either transcribed at a low level (such as amylase) or they could have another function, including a role in metabolic pathways, molecular signaling or transport. These results suggested that these genes may be transcribed for another reason, other than bacterial defense in an axenic medium (Table S6).

### 3.8. Ecological Context

**Figure 6:**
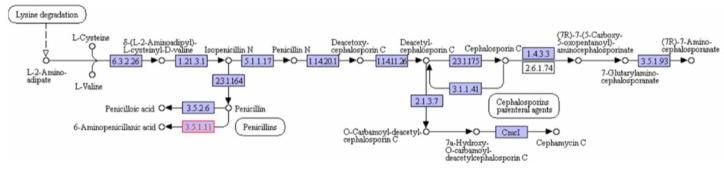
Representation of metabolic pathway performed on KEGG website. The map shows the pathway of secondary metabolite biosynthesis. In red color are represented the enzymes found in transcriptome and proteome of *W. magna*.

Amoebae cope with a variety of microorganisms in the environment. To survive and defend themselves, they produce a repertoire of several classes of proteins. We identified transcripts and proteins involved in various metabolic pathways related to the biosynthesis of secondary metabolites (Figure S5 and Table S7). Among them was a penicillin amidase transcribed at low level. This enzyme serves for the biosynthesis of penicillin derivatives (Figure 6).

We also identified two genes with multidomains related to polyketide synthases producing natural metabolites with antimicrobial properties [66,67] (Figure S5). Among them, we found a gene encoding polyketide synthase containing ketide synthase, acyltransferase, thioesterase, enoyl reductase and ketoreductase domains (Figure S5). This gene, sharing the best hit with a *N. gruberi* gene, was expressed at a high level. Interestingly, several transcripts (n = 33) and proteins (20) were related to xenobiotic biodegradation and metabolism, encompassing 2-haloacid dehalogenase (K01560) and alkane 1-monooxygenase (K00496), which are enzymes specifically involved in chloroalkane and chloroalkene degradation and in caprolactam degradation, respectively (Table S2). These enzymes have crucial functions in the degradation of persistent organic pollutants, which cause substantial problems for the environment [68]. Moreover, we identified the presence of ATP-binding cassette (ABC) transporters that constitute a protein family involved in the import and export of a large variety of substrates and in the regulation of several cellular processes, such as chemotaxis [69]. These transporters could have played the role of detoxifiers by exporting xenobiotics and endogenous secondary metabolites [70]. Furthermore, we found several enzymes involved in the metabolism of terpenoid compounds, such as terpene and squalene, many of which have pharmaceutical or biological properties [71] (Table S7).

## 4. Discussion

This study analyzed for the first time the transcriptome and proteome of *W. magna*. It provides unique data on the molecular mechanisms and metabolic pathways used by *W. magna* C2c maky during axenic mass culture. Firstly, high throughput RNA-sequencing technology was carried out to obtain a full set of transcripts of the amoeba in bioreactor culture condition. A similar dataset was generated for the transcriptome of *Naegleria fowleri* [72] performed with Illumina HiSeq 2000 technology. Among the 8,790 transcripts, 70.3% matched with known proteins in the nr database. The absence of matches in the nr database for the remaining transcripts was probably due to the currently limited set of sequenced amoebal genomes. Indeed, only the genomes of *A. castellanii*, *Acanthamoeba triangularis, Vermamoeba vermiformis, Balamuthia mandrillaris* and four genomes of the *Vahlkampfiidae* family (*W. magna*, *N. gruberi*, *N. fowleri* and *Naegleria lovaniensis*) were sequenced and analyzed [28,32,72,73,74,75,76,77].

Based on and completing genomic and transcriptomic data, the proteomic analysis provided a better description and understanding of the molecular and physiological processes of the amoeba. The proteome was performed from two independent bioreactor samples each divided into eight fractions. The analysis allowed 3561 proteins to be identified of which a very large majority (3376; 95%) had an assigned function in nr. To obtain a detailed and complete characterization into gene function, we explored the different classification systems. The COG and KEGG classification uncovered that the transcripts and proteins are involved in various metabolic pathways and diverse molecular mechanisms.

For transcripts and proteins, the top five enriched COG categories were similar, although they presented in a different order. Furthermore, we observed that the proportion of proteins assigned to the top five COG categories (“unknown function”, “post-translational modification, protein turnover, chaperones”, “signal transduction mechanisms”, “intracellular trafficking secretion and vesicular transport” and “translation, ribosomal structure and biogenesis”) was higher than the proportion for the transcripts. Furthermore, KEGG-based classification reported a large proportion of enzymes involved in the metabolism of carbohydrates, amino acids and lipids. No apparent differences were observed between metabolic pathways identified through the analyses of the transcriptome and the proteome. This suggests that the proteomic data mainly confirmed here the transcriptomic data. Although the data of the transcriptome and the proteome are congruent, we noted some differences such as the high expression of the Nox2 gene, whereas the protein was not identified within the proteomic data. These differences between the transcriptomics and proteomics data have already been widely described in the literature. Indeed, these differences can be explained due to several reasons, as the detection of RNA-seq is a much more sensitive method than the determination of the proteome [78]. Moreover, the regulatory processes which can occur after transcription of mRNA, including post-transcriptional, translational, post-translational and protein degradation regulation mechanisms, as well as the half-life of RNA and of the corresponding proteins, could be another reason for the difference between the transcriptomics and proteomics results [31,79,80]. The cytoskeleton allows cellular “scaffolding”, which is essential for cell motility, intracellular transport and architectural structure [81]. Generally, amoebae adhere to a support on which they adopt a particular morphology with pseudopodia presence and move by an amoeboid movement [58,82]. Our transcriptomic and proteomic data revealed multiple transcripts (349) and proteins (151) related to cytoskeleton. In a previous study, the analysis of the *W. magna* genome revealed 625 genes related to cytoskeleton, which represented 3.4% of the gene repertoire [28]. This proportion of genes related to cytoskeleton is greater than for *Naegleria* species, which could be related to the high mobility of *W. magna*. In our COG analysis, we found that only 1.5% of genes related to cytoskeleton were expressed, and 0.5% genes were translated. These low proportions could suggest that the amoebae cultivated in a bioreactor (suspension mode) require a structural organization of the cytoskeleton different from that of amoebae grown on a support (adherence mode). Microtubules and microfilaments were found in the present work to be essential structural components of the *W. magna* cytoskeleton. Under specific conditions, *W. magna* has a flagellate stage, as is for the case of *Naegleria* species [5]. In our study, the identification of microtubule-related proteins involved in flagellate formation indicates that *W. magna* could temporarily move in its flagellated form during bioreactor growth.

In a large-scale laboratory fermenter, *W. magna* uses carbohydrates, lipids and amino acids through the Krebs cycle and a branched respiratory chain with oxygen as final electron acceptor. Although considered to be fully aerobic, the identification of transcripts and proteins involved in anaerobic respiration shows that this amoeba has the ability to adapt to low oxygen concentrations. This parallels previous findings for *A. castellanii*, *N. gruberi* and other organisms regarding the versatility of metabolism and the possibility of a facultative, anaerobic metabolism [32,33,35,83]. For some protists living under low-oxygen condition, the anaerobic respiration takes place in mitochondrion-related organelles named hydrogenosomes [84]. In our study, we identified N-terminal mitochondrial transit peptides for two proteins related to anaerobic respiration. The same result was predicted by bioinformatic for *N. gruberi* anaerobic protein, with which *W. magna* shared best hits [32]. However, an in vitro study showed that FeFe-hydrogenase and its associated maturase is located in cytosol [85]. These results suggest that the FeFe-hydrogenase and its associated maturase in *W. magna* are likely located in mitochondria or in cytosol.

The use of such facultative anaerobic metabolism could indicate that some cells were not oxygenated enough. Therefore, the detection of proteins involved in *W. magna* metabolism versatility may provide us with an indication of parameters that could be modified in order to improve growing conditions. Free-living amoebae have a predatory role in the control of bacterial populations in soils and aquatic environments [6,7,86,87]. To feed on bacteria, amoebae have a set of genes that set up a variety of molecular mechanisms to destroy bacteria [33,88]. Several years ago, we cultivated *W. magna* C2c maky in axenic medium, in which the amoebae lived without the presence of bacteria and only by the absorption of dissolved nutrients. However, the identification of transcripts and proteins related to the degradation and phagocytosis of bacteria suggests that *W. magna* still has the ability to feed on bacteria. This hypothesis was verified by plating *W. magna* C2c maky cultivated on non-nutrient agar plates seeded with *E. coli* (data not shown). This is an important feature for this amoeba, which is produced in bioreactors with the objective to decontaminate cooling towers from legionella. The expression of these proteins may be necessary for the degradation of nutrients in the growth medium. Similar results were obtained in a *N. gruberi* metabolism study, in which Opperdoes et al. reported that the *N. gruberi* strain NEG-M, cultivated for many years in an axenic medium, had retained all the genes involved in bacterial degradation within the genome [33]. Moreover, we found the sequences related to the bacterial degradation in the transcriptome of *N. gruberi* strain NEG-M [72]. However, all these genes related to bacterial defense mechanisms have another function, which could explain the reason for the expression of these genes in the absence of bacteria. In the environment, amoebae live in communities with other microorganisms and face many complex ecological challenges [7]. Indeed, they are in constant competition with fungi, bacteria, protists and other amoebae for food and multiplication. Furthermore, amoebae must deal with predation and toxins [70]. To survive, *W. magna* seems to harbor an ancient weapon in the defense against the growth of other microorganisms. Analysis reported the presence of some enzymes involved in the biosynthesis of secondary metabolites such as a penicillin amidase or polyketides synthase, which may allow it to defend itself against the microbial communities in the environment [7,70]. However, penicillin amidase is also found in many different microorganisms that appear to use this enzyme for other purposes such as the assimilation of carbon source [89]. *A. castellanii* has demonstrated potent bactericidal properties against Methicillin-resistant *Staphylococcus aureus* (MRSA) [90]. A large repertoire of putative polyketide synthases has been identified in the *D. discoideum* genome [91]. Therefore, we can hypothesize that amoebae could be potential candidates as a new source of antimicrobial compounds. Furthermore, we have identified transcripts and proteins of *W. magna* that support a potential involvement in the degradation of xenophobic compounds. These man-made compounds cause considerable problems to the environment because they are often refractory to degradation [68,92]. As for some bacteria, *W. magna* could be tested for the control of the degradation of persistent organic chemicals [93].

RNA sequencing has a broad range of applications allowing several scientific questions to be responded to such as the composition of transcriptome or comparison of gene expression profiling between different samples [94]. Biological replicates are essential for differential expression analyses in interpretation of the quantification of the level of gene expression [95,96,97]. To analyze the composition of transcriptome, they are less important as they are only used to overcome natural biological variability. Association with proteome analysis, done in duplicate in the present study, overcomes this problem. Thus, we could identify the proteins for nearly all transcripts detected, even those with a limited number of reads. This is likely because our RNA-seq study included a high number of reads and coverage [98]. Indeed, we obtained 217 million reads from RNA sequencing, which is efficient for a transcriptomic study [72,95].

## 5. Conclusions

The study provides new insight in the understudied amoebic field. Overall, our transcriptomic and proteomic survey expands the still limited knowledge on FLAs, which are common in the environment. These analyses provide new insights into the metabolic pathways and biological processes of *W. magna* cultivated using untraditional methods. Moreover, the identification of enzymes associated with secondary metabolite pathways suggests that *W. magna* could have the capacity to produce and export compounds with antimicrobial activity.

## Data Availability

The transcriptome data are deposited in EBI-EMBL under bioproject number PRJEB30797. The proteome generated during the current study are deposited in ProteomeXchange via the PRIDE database under the project accession number PXD016724.

## Supplementary Materials

The following are available online at https://www.mdpi.com/2076-2607/8/5/771/s1, Figure S1: Representation of taxonomic distribution of protein assigned to a function in nr database, Figure S2: Phylogenetic tree for *W. magna* [FeFe] hydrogenase maturation protein, Figure S3: Phylogenetic tree for *W. magna* [FeFe] hydrogenase maturation protein, Figure S4: Phylogenetic tree for [FeFe]-hydrogenase maturation protein HydG and Figure S5: Representation of conserved domain of beta-ketoacyl synthase; Table S1: Transcriptomic and proteomic annotation, Table S2: Proteomic data, Table S3: KEGG annotation, Table S4: Motility study, Table S5: Metabolism study, Table S6: Mechanism defense study and Table S7: Secondary metabolism study.
