# Supplementary material for: Insight into the Lifestyle of Amoeba Willaertia magna during Bioreactor Growth Using Transcriptomics and Proteomics
Source: Microorganisms. 2020 May 21;8(5):771. doi: 10.3390/microorganisms8050771 (PMC7285305; doi:10.3390/microorganisms8050771)
Supplement: Supplementary file 1 [file microorganisms-08-00771-s001.zip › Supplementary_Material_Figure.docx]

**Supplementary Material Figure**

**Insight into the amoeba *W. magna* life style in bioreactor growth by transcriptomic and proteomic analysis.**

**Issam Hasni^1,2,3^, Philippe Decloquement^1^, Sandrine Demaneche^2^, Mouh Rayane Mameri^2^, Olivier Abbe^2^_,_ Philippe Colson^1,3^ and Bernard La Scola^1,3^***

**Affiliations :** ^1^Aix-Marseille Univ., Institut de Recherche pour le Développement IRD 198, Assistance Publique – Hôpitaux de Marseille (AP-HM), Microbes, Evolution, Phylogeny and Infection (MEΦI), UM63 ; ^2^R&D department, Amoéba, France ; ^3^Institut Hospitalo-Universitaire (IHU) - Méditerranée Infection, France,

[issemhasni@gmail.com](mailto:issemhasni@gmail.com) (I.H.); [Philippe.DECLOQUEMENT@univ-amu.fr](mailto:Philippe.DECLOQUEMENT@univ-amu.fr) (P.D.); [Sandrine.DEMANECHE@amoeba-biocide.com](mailto:Sandrine.DEMANECHE@amoeba-biocide.com) (S.D.); [mameri77@yahoo.fr](mailto:mameri77@yahoo.fr) (M.M.); [Olivier.Abbe@amoeba-biocide.com](mailto:Olivier.Abbe@amoeba-biocide.com) (O.A.); [philippe.COLSON@univ-amu.fr](mailto:philippe.COLSON@univ-amu.fr) (P.C.) ; [bernard.la-scola@univ-amu.fr](mailto:bernard.la-scola@univ-amu.fr) (B.L.).

*** Corresponding author :** Bernard La Scola, MD, PhD, Institut Hospitalo-Universitaire (IHU) -Méditerranée Infection, France, 19-21 Boulevard Jean Moulin, 13385 Marseille Cedex 05, France. Telephone: +33 4 91 32 43 75, Fax: +33 4 91 38 77 72, E-mail: bernard.la-scola@univ-amu.fr.

**Supplementary Figure**


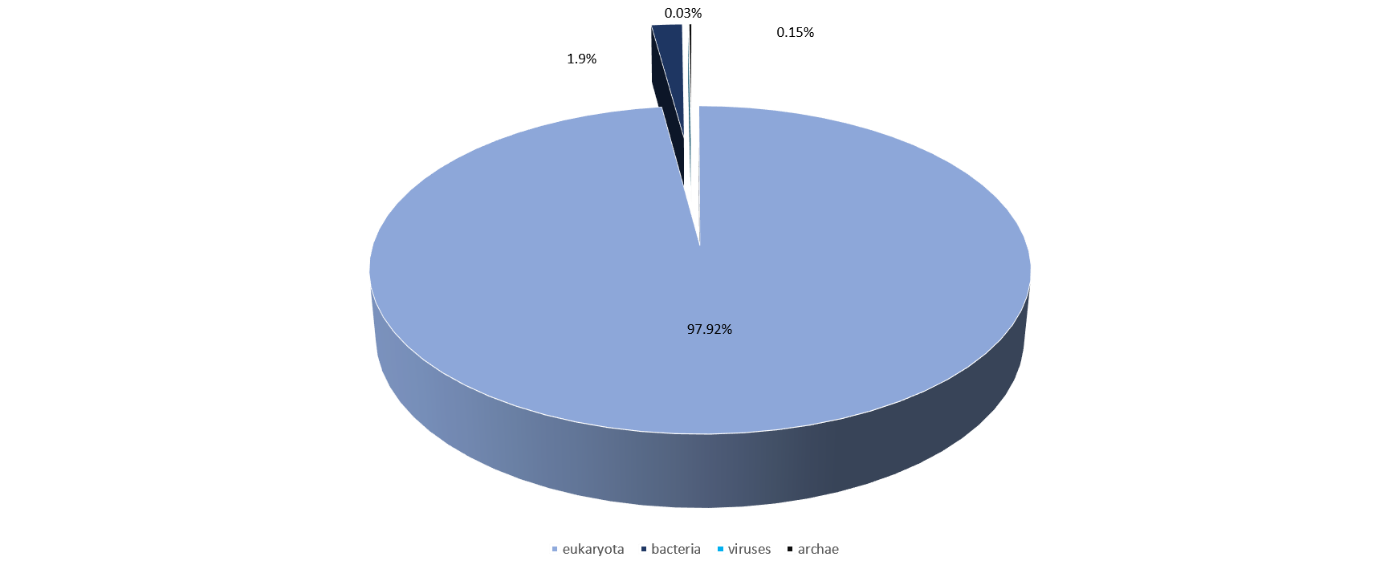


**Figure S1:** Representation of taxonomic distribution of protein assigned to a function in nr database.


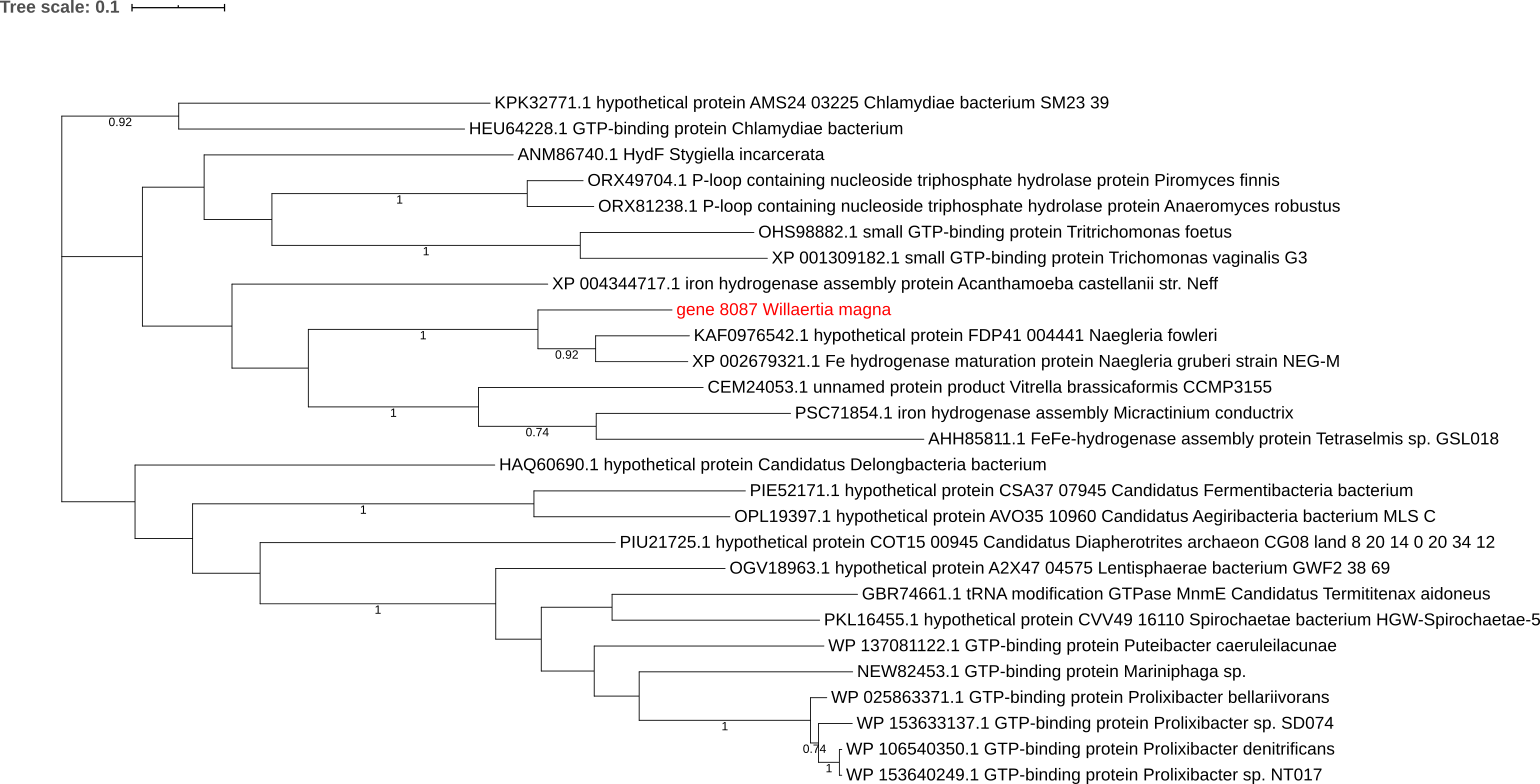


**Figure S2:** Phylogenetic tree for *W. magna* [FeFe] hydrogenase maturation protein.


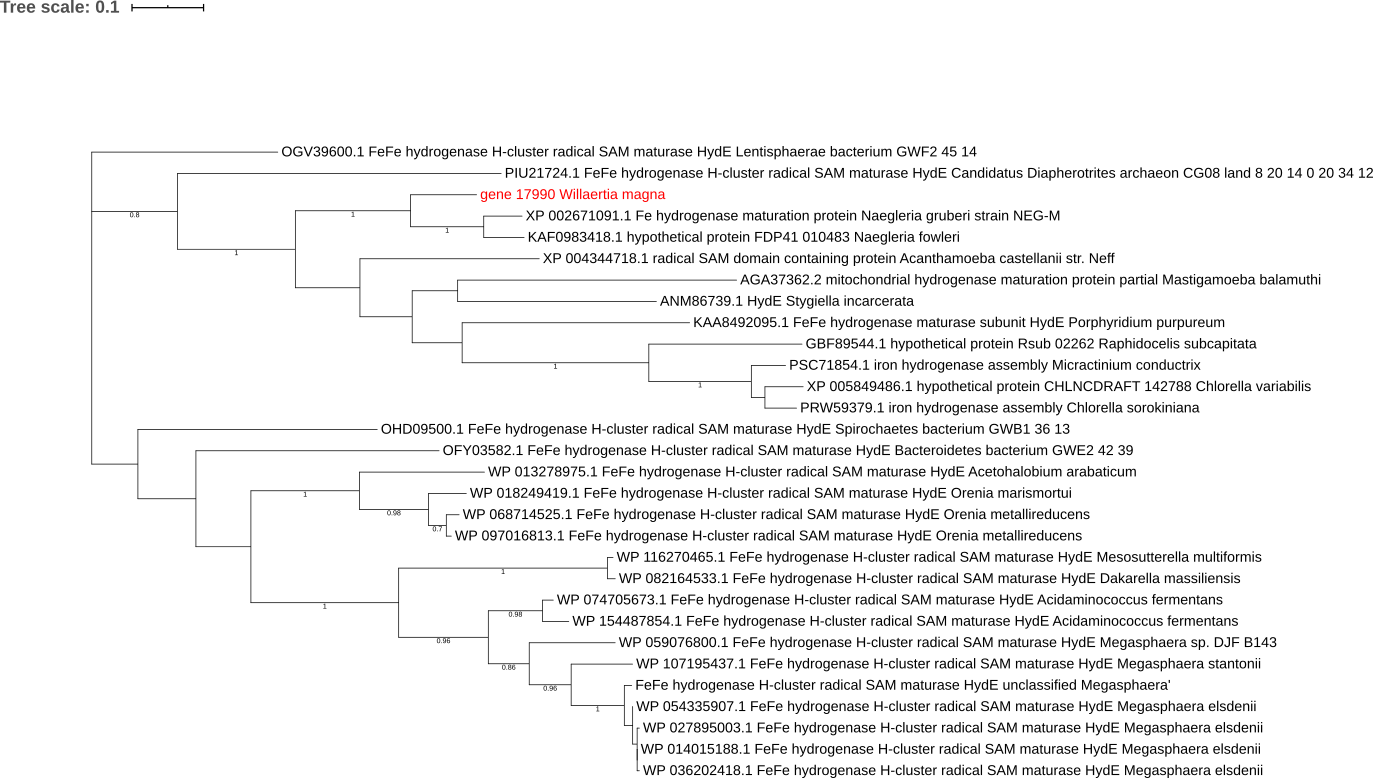


**Figure S3:** Phylogenetic tree for *W. magna* [FeFe] hydrogenase maturation protein.


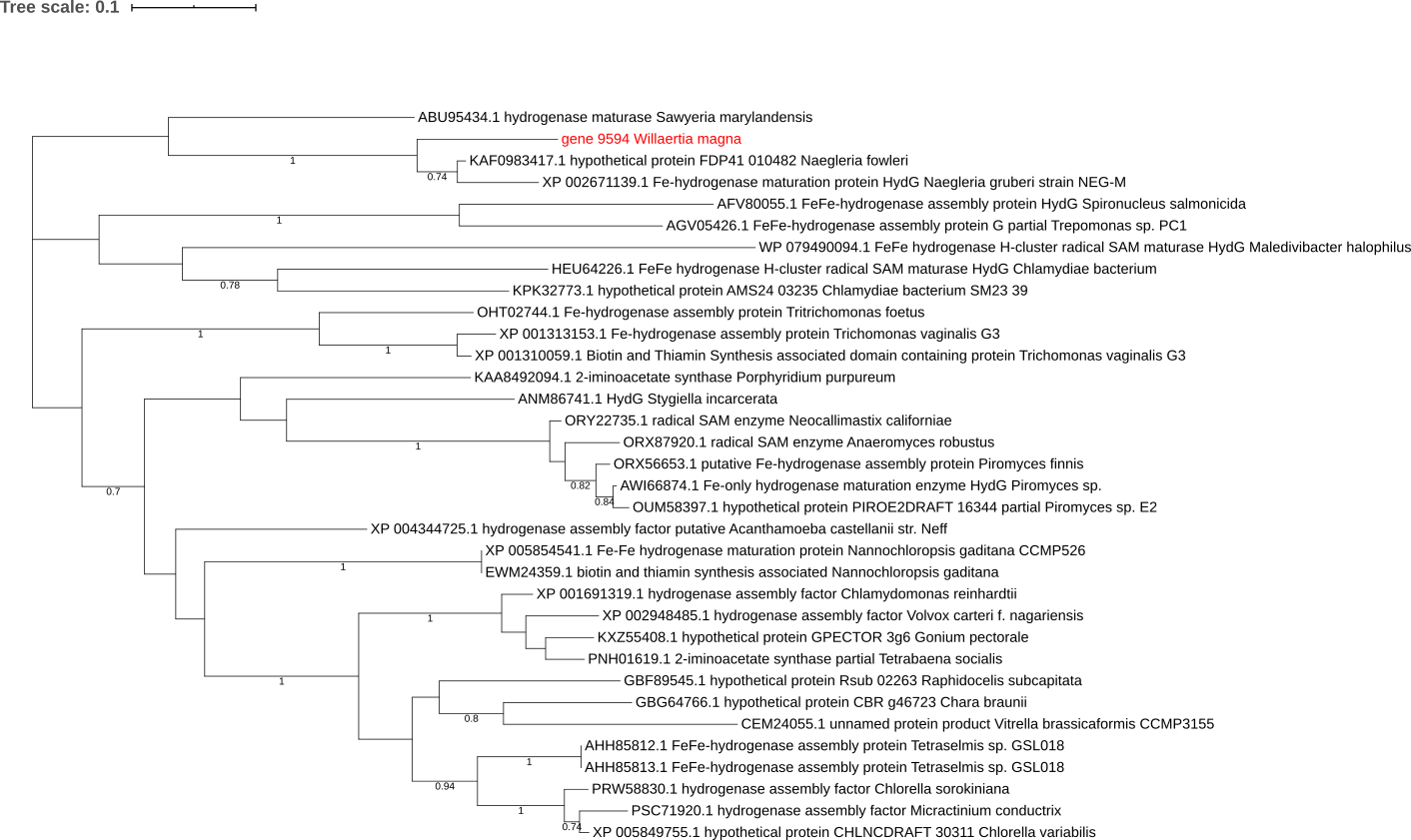


**Figure S4:** Phylogenetic tree for [FeFe]-hydrogenase maturation protein HydG


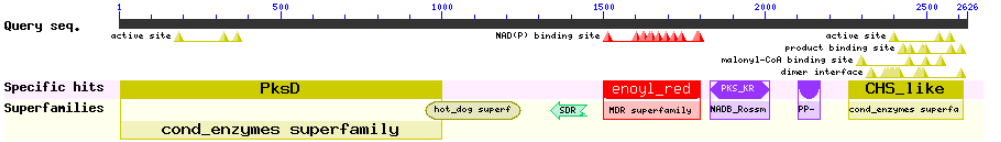


**Figure S5:** Representation of conserved domain of beta-ketoacyl synthase. The analysis was performed by protein comparison against CDD database using the NCBI conserved domain website (<https://www.ncbi.nlm.nih.gov/Structure/cdd/wrpsb.cgi>). The characteristics of the conserved domain was reported as following: PksD domain; Acyl transferase domain in polyketide synthase (PKS) enzymes, Enoyl red domain; enoyl reductase of polyketide synthase; Putative enoyl reductase of polyketide synthase, CHS-like domain; Chalcone and stilbene synthases; plant-specific polyketide synthases (PKS) and related enzymes, PKS KR domain; this enzymatic domain is part of bacterial polyketide synthases.It catalyses the first step in the reductive modification of the beta-carbonyl centres in the growing polyketide chain. It uses NADPH to reduce the keto group to a hydroxy group, hot-dog super family; the hotdog fold was initially identified in the Escherichia coli FabA, PP-binding; Phosphopantetheine attachment site; A 4'-phosphopantetheine prosthetic group is attached through a serine, SDR super family; Short-chain dehydrogenases/reductases (SDR); SDRs are a functionally diverse family of oxidoreductases that have a single domain with a structurally conserved Rossmann fold (alpha/beta folding pattern with a central beta-sheet), an NAD(P)(H)-binding region, and a structurally diverse C-terminal region.
